# Perspectives of bereaved relatives of patients with haematological malignancies concerning preferred place of care and death: A qualitative study

**DOI:** 10.1177/0269216318824525

**Published:** 2019-01-30

**Authors:** Dorothy McCaughan, Eve Roman, Alexandra G Smith, Anne C Garry, Miriam J Johnson, Russell D Patmore, Martin R Howard, Debra A Howell

**Affiliations:** 1 Epidemiology and Cancer Statistics Group, University of York, York, UK; 2 Department of Palliative Care, York Hospital, York, UK; 3 Wolfson Palliative Care Research Centre, University of Hull, Hull, UK; 4 Queen’s Centre for Oncology and Haematology, Castle Hill Hospital, Hull, UK; 5 Department of Haematology, York Hospital, York, UK

**Keywords:** Leukaemia, lymphoma, multiple myeloma, preferred place of care, preferred place of death, qualitative research

## Abstract

**Background:**

People with haematological malignancies have different end-of-life care patterns from those with other cancers and are more likely to die in hospital. Little is known about patient and relative preferences at this time and whether these are achieved.

**Aim:**

To explore the experiences and reflections of bereaved relatives of patients with leukaemia, lymphoma or myeloma, and examine (1) preferred place of care and death; (2) perceptions of factors influencing attainment of preferences; and (3) changes that could promote achievement of preferences.

**Design:**

Qualitative interview study incorporating ‘Framework’ analysis.

**Setting/participants:**

A total of 10 in-depth interviews with bereaved relatives.

**Results:**

Although most people expressed a preference for home death, not all attained this. The influencing factors include disease characteristics (potential for sudden deterioration and death), the occurrence and timing of discussions (treatment cessation, prognosis, place of care/death), family networks (willingness/ability of relatives to provide care, knowledge about services, confidence to advocate) and resource availability (clinical care, hospice beds/policies). Preferences were described as changing over time and some family members retrospectively came to consider hospital as the ‘right’ place for the patient to have died. Others shared strong preferences with patients for home death and acted to ensure this was achieved. No patients died in a hospice, and relatives identified barriers to death in this setting.

**Conclusion:**

Preferences were not always achieved due to a series of complex, interrelated factors, some amenable to change and others less so. Death in hospital may be preferred and appropriate, or considered the best option in hindsight.

**What is already known about the topic?**

- Home is often considered the preferred place of care and death for most people.
- Hospital deaths occur more often in patients with haematological than other cancers.
- Relatives’ accounts of their own and decedents’ preferences, and their reflections on the factors influencing their achievement, are unexplored in haematological malignancies.

**What this paper adds?**

- This is the first study to examine preferred place of care and death in patients with blood cancers from the perspectives of bereaved relatives.
- Factors impacting on achievement of preferences were disease characteristics, the occurrence and timing of end-of-life discussions, family networks and resource availability.
- Hospital was sometimes preferred and, on reflection, some relatives identified this as the ‘right’ place for the patient to have died.

**Implications for practice, theory or policy**

- Early, honest and realistic communication of risk and uncertainty, initiated by haematologists, could prevent over-optimism and facilitate advance planning among patients and relatives, as well as allowing primary care staff adequate time to prepare for the patient’s potential death at home.
- Standardisation of hospice policies could result in this service being better able to meet the needs of patients with haematological malignancies.
- Relatives are key members of the caregiving team; their reflections were new, insightful and important.

## Introduction

Haematological malignancies are complex cancers broadly categorised as leukaemias, lymphomas and myeloma; they affect all age groups, but are common in older people.^1^ Collectively, these diseases represent around 8% of all new cancers diagnosed annually in the United Kingdom (UK).^2^ They can be highly aggressive or indolent, with treatment varying in intensity and being given for differing purposes.^3^ For example, chemotherapy may be administered as a course (i.e. with a specific number of cycles); it can be given intermittently (e.g. at times of relapse/progressive disease), or constantly (e.g. daily without a planned end date); and it can be given to induce and maintain remission, for disease control, or to improve quality of life.

Despite significant treatment advances, many haematological malignancies remain incurable, have unpredictable and uncertain trajectories, and highly variable outcomes that are particularly poor for some subtypes.^4–6^ Deterioration may be unexpected and sudden or gradual, as response to chemotherapy diminishes. This can complicate decision-making regarding treatment cessation, initiation of end-of-life discussions and ascertainment of care preferences.

End-of-life pathways are recognised to differ for patients with haematological malignancies from other cancers. People with blood cancers, in the United Kingdom and elsewhere, are more likely to receive hospital-based intensive treatments close to the end of life;^7,8^ less likely to receive palliative and/or hospice care,^9,10^ or to receive this closer to death, when they are seriously ill;^11,12^ and more likely to die in hospital than at home or in a hospice.^13–15^

Studies indicate that most people would prefer to die at home^16,17^ and that their family caregivers often support this decision.^18^ However, such assertions are increasingly being questioned. A recent systematic review of the UK literature, for example, highlights the scale and impact of missing data in studies about place of death and the resulting lack of clarity around preference for home death.^19^ Another study, based on interviews with 59 bereaved family carers of older people dying at home, revealed the complex and often overpowering emotions that may be associated with home death, which led the authors to question if this setting is necessarily better than the others.^20^ Other interviews, exploring end-of-life hospital use by patients with palliative care needs, found that beyond treatment patients also appreciated being cared for by staff with expert knowledge of their condition, as this made them feel ‘safe’ and brought relief for family members.^21^ Symptom outcomes are also said to be better for individuals dying in hospital, compared to home.^22^

To date, little is known about preferred place of care and death in patients with haematological cancers and their families. We therefore conducted interviews with bereaved relatives of patients with these diseases, one strand of a larger qualitative study that included interviews with haematology doctors and nurses, GPs and palliative care clinicians.^23,24^ The aim of the interviews with relatives was to explore (1) preferred place of care and death; (2) reflections on experiences following the patient’s death, including perceptions of factors influencing the attainment of preferences; and (3) changes that could promote achievement of preferences.

## Methods

We used qualitative methods (in-depth interviews), described below in accordance with the consolidated criteria for reporting qualitative studies (COREQ).^25^

### Setting and participants

The study was set within the United Kingdom’s Haematology Malignancy Research Network (HMRN: www.hmrn.org), a collaborative programme of work involving researchers and clinical staff, which was established in 2004 to generate evidence to improve patient care,^26^ using various methodologies, including qualitative research. For the present project, haematologists at a single hospital in the HMRN area purposively identified the main bereaved family care provider(s) of patients with leukaemia, lymphoma or myeloma, who had died within the previous 2 years (maximum). Potential interviewees were then sent an information sheet about the study and an invitation to take part. Inclusion was not limited by place of death or end-of-life preferences and whether these had been met.

Individuals who wanted to take part were asked to contact a researcher (D.A.H.) to arrange an interview. In total, 13 relatives were approached and 10 agreed to be interviewed (Table 1). Sample size was determined ethically and pragmatically. Due to the sensitive nature of the topic, we did not want to conduct more interviews than necessary, nor did we wish to overburden the haematologists facilitating recruitment. Data saturation (when no new or relevant information is forthcoming^27^) was, therefore, deemed unlikely and so was not an aim of the study.

**Table 1.** Characteristics of relative participants and decedents.

Study ID	Participant characteristics		Decedent characteristics				Months between patient’s death and relative’s interview
	Relationship to decedent	Gender	Diagnosis	Age at death (years)	Gender	Survival (months)	
R1	Spouse	F	Leukaemia	67	M	6	10
R2	Daughter	F	Myeloma	Unknown	F	Unknown	Unknown
R3	Spouse/Daughter	M /F	Lymphoma	81	F	22	3
R4	Spouse	F	Myeloma	75	M	8	21
R5	Spouse	F	Lymphoma	71	M	4	5
R6	Spouse	F	Lymphoma	80	M	51	9
R7	Spouse	F	Lymphoma	74	M	18	7
R8	Spouse	F	Leukaemia	66	M	93	9
R9	Spouse	M	Leukaemia	66	F	31	4
R10	Spouse	F	Leukaemia	69	M	5	6

### Data collection and analysis

Written consent was obtained before each interview and assurances given concerning confidentiality and anonymity. Interviews were conducted in 2014 (eight in relatives’ homes and two in a private university office), with the aid of a semi-structured topic guide (Table 2), sufficiently flexible to incorporate new lines of inquiry. Interviews lasted up to 90 min and were audio-recorded and transcribed verbatim.

**Table 2.** Topic guide.

• Discussions regarding broad end-of-life issues (treatment failure/cessation, prognosis) • Discussions regarding preferred place of care and death, and changes over time • Was preferred place of care and death achieved? If not why not • Factors considered to have prevented/promoted death at home/hospital • Key changes that could facilitate care and death in the preferred place

The ‘Framework’ method was used to analyse the data.^28^ Framework is a flexible tool, not aligned with a particular epistemological, philosophical or theoretical stance, but adaptable to various qualitative approaches aiming to generate themes. It facilitates the identification of commonalities and differences in qualitative data, focusing on the relationships between different data segments, with the aim of drawing descriptive and/or explanatory conclusions clustered around themes.^29^ Our analytical approach was predominantly deductive; that is to say, it was guided by the research questions, derived from the relevant literature. It followed the systematic sequential steps: data familiarisation (by reading and re-reading transcripts); identification of codes and development of a coding scheme that was applied to individual transcripts, expanded and modified as necessary to accommodate data; aggregation of codes (units of meaning) and theme development; summarising and charting of data (using electronic spreadsheets to facilitate comparison within and between cases); and cross-comparison of themes throughout the dataset to promote synthesis and interpretation.^28^ Negative or ‘deviant’ cases were sought in the data and used to develop and refine the analysis.^30^

Interviews were conducted by D.A.H. and data analysis was led by D.M., with guidance from D.A.H. D.M. and D.A.H. (both former registered nurses and experienced qualitative researchers) regularly discussed and refined codes and emerging themes. Disagreements facilitated refinement of the analysis.^31^ An independent researcher (R.H.) assessed the ‘fit’ of the coding scheme of two interviews,^32^ confirming that the strategy was comprehensive and appropriate.^33^

#### Ethical approval

National Health Service (NHS) ethical approval was obtained from Yorkshire and The Humber Research Ethics Committee (REC: 11/YH/0306).

## Results

The characteristics of interviewees are shown in Table 1. More decedents were men than women; more women than men were interviewees and these were mainly spouses, with the exception of two daughters. We included relatives whose family members had had leukaemia, lymphoma and myeloma, with patients’ ages ranging from 66 to 81 years and survival spanning 4–93 months. Most relatives were interviewed within 3–10 months of the patient’s death. Results are presented (with verbatim quotations), according to three key themes.

### Theme 1: preferred place of care and place of death

Relatives discussed their own and the patient’s preferred and actual place of care and death (Table 3). Preferences changed over time, with differing views sometimes described between patients and their relatives. Home was the most common preference, then hospice; no patients, and only one interviewee (R4), were inclined towards hospital death. Six patients died at home, four in hospital and none in hospice.

**Table 3.** Patients’ (reported) and relatives’ (expressed) preferred place of death, changes over time and actual place of death.

Study ID	Patient’s PPD	Relative PPD	Patient’s change of preference	Relative’s change of preference	Actual place of death
R1	Home	Hospice	He ‘couldn’t have cared’ (after sudden deterioration)	Hospital death was ‘very peaceful’ and care was good	Hospital
R2	Hospice	Home	Home	Unchanged	Home
R3	Not stated	Not stated	Not stated	Not stated	Home
R4	Not stated	Hospital	Not stated	Not stated	Hospital
R5	Hospice	Home	Not stated	‘With hindsight, hospital was the right place’ for husband to die (after sudden deterioration)	Hospital
R6	Not stated	Home	Not stated	Unchanged	Home
R7	Home	Home	Unchanged	Unchanged	Home
R8	Home	Home	Unchanged	Unchanged	Home
R9	Home	Home	Unchanged	Unchanged	Home
R10	Home	Unsure	Not stated	Hospital was the ‘right place’ (for husband to die)	Hospital

PPD: preferred place of death.

Several relatives (R7, R8, R9) reported sharing patients’ strong preference for home care and death, which remained unchanged as the patient’s condition declined. Death anywhere but home was considered highly undesirable by these interviewees, who portrayed themselves as advocates, prepared to ‘speak up’ to ensure patients died at home:

he wanted to stay at home … I wasn’t going to have him go in [to hospital] because he didn’t want to … the night he was dying he was getting restless and I said ‘will you let me get the doctor …’, ‘No, no, they’ll take me [to hospital] …’ and I said ‘no, they won’t …’ I wouldn’t have let him go back in … (R7)

Hospice was cited as the ‘fall back’ option for some people whose first preference was for death at home. One relative (R1) said she had wanted her husband to die in hospice to avoid future painful associations of him having died in the family home:

she was very clear that she wanted to die at home, and if she couldn’t, then it would have been in the hospice if at all possible, she absolutely did not want to be in hospital. (R9)

I didn’t want him to die at home … it would have brought too many memories … I would have been quite happy for him to go into hospice. (R1)

R5’s husband had expressed a preference for hospice care, though her own inclination had been for him to die at home. However, when her husband developed distressing symptoms, he was urgently admitted to hospital, where he died:

he wanted the final chapter of his life to be in the hospice … he didn’t want to die in hospital … but he was coughing up blood and couldn’t get his breath. (R5)

Only one participant (R4) indicated having held a preference for her spouse to receive end-of-life care in hospital, due to anxiety about her ability to provide adequate care at home. Her preference was for her husband to receive professional support in a nearby hospital, where she could visit without restriction. Reflecting on his death, this interviewee noted the high-quality care her husband had received during his final weeks, which had convinced her that death in hospital had been appropriate:

the hospital was half a mile away … I could be there in five minutes … they made us feel totally involved and welcomed … the staff nurses and nursing assistants were wonderful … For us hospital was the right place, if we’d been at home we would have been stressed … that we weren’t doing the right thing … I was very grateful for that professional support … he was needing two people to help him go to the loo … he was very weak … I don’t know how I could have managed at home without someone with me. (R4)

Similarly, several relatives (R1, R5, R10), whose spouses had not wished to die in hospital but had done so, stated that with hindsight they had come to view the patient’s death in hospital as appropriate and acceptable, partly because (re)admission had brought expert nursing care and symptom relief. These relatives suggested that they would have struggled to care for the patient at home, particularly overnight, when assistance from community nurses could be unreliable. However, a sense of failure associated with relinquishing responsibility for caring for their spouse was apparent in some accounts:

I felt I’d failed him in some way with him having to go back in [to hospital], but I know he was really frightened when he couldn’t take his breath during the night … and he felt a lot safer on the ward where he could get help incredibly quickly … the support we got couldn’t have been better … in the end it was the right place. (R5)

Place of death was reported as less significant to some patients in their final hours than feeling safe and secure in a familiar environment, cared for by people they knew and trusted. One relative (R1), whose husband had wanted to die at home, described his death in a small community hospital as ‘very peaceful’:

those last 48 hours, I don’t think he really cared [where he was] … there was something about [hospital] that made it more user friendly … it was small, it was more intimate … it was less clinical and he liked the staff … he didn’t like a big [hospital]. (R1)

Contrastingly, enabling home death appeared vitally important to relatives for whom dying at home was intrinsic to their notion of a ‘good death’. R2, for example, expressed a desire to ‘give’ her mother a good death at home. As a nurse, she said she had felt competent to manage her mother’s symptoms and needs at home, with support from family and community nursing services. This relative recounted positive memories of her mother’s ‘lovely’ death, which she felt had drawn family members together:

she [mother] had a good death at home … it was lovely … even when she was breathing her last we were ringing this friend of hers saying ‘do you want to come round and say goodbye’. (R2)

### Theme 2: factors perceived to influence achievement of preferred place of care and death

**Figure 1.**
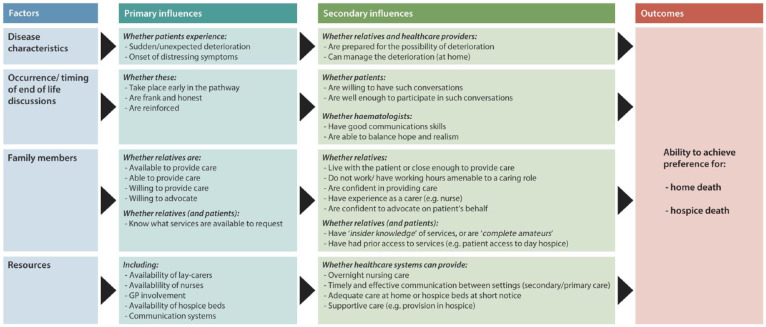
Factors perceived to impact on achievement of preferred place of care and death (home or hospice).

Four inter-related factors were perceived as influencing achievement of preferences: disease characteristics, the occurrence and timing of end-of-life discussions, family networks and resource availability. These are summarised in Figure 1.

#### Disease characteristics

A major obstacle relatives identified to attainment of preferences was the unpredictability of the patient’s disease. Sudden deterioration, alongside the onset of distressing symptoms, resulted in relatives contacting haematology services, the General Practitioner (GP) or out-of-hours medical services, for advice and assistance. Consequently, some patients previously discharged home to die were readmitted to hospital, where they subsequently died. Such events were said to have occurred even when there was input from community palliative care nurses:

with this kind of illness, events overtake themselves, they [patients] have a downturn and they need immediate attention and your first port of call is the hospital … they have this raging temperature and they need immediate medical assistance … antibiotics and blood transfusions, fluids, so you ring the helpline and they get you in. (R1)

Sudden decline in the patient’s condition could mean that discharge home, or hospice transfer (from hospital), was not always desirable or feasible at this time:

it would have been really cruel to move him when he suddenly went downhill that quickly … he was far better supported by people who really knew him instead of him having to travel [to hospice] when he was feeling terrible … to a situation where we didn’t know anybody. (R5)

#### Occurrence and timing of discussions about treatment cessation, prognosis and place of care and death

The occurrence and early introduction of end-of-life discussions appeared important for meeting preferences for home death, particularly if care-packages required organising to facilitate this. Most relatives recalled at least one discussion with a haematologist concerning treatment cessation in the months/weeks preceding the patient’s death; they recalled being told that there was ‘nothing more to be done’ in terms of treatment and that they had ‘run out’ of options. Some people commented that it had been clearly explained at diagnosis that the effectiveness of treatment would diminish over time, a message reinforced in follow-up consultations:

when the cancer originally happened [haematologist] gave a percentage … [chance of] cure … and then when [patient] went back … it was definitely not such a good recovery rate … [Haematologist] did tell us that before [patient] even started the chemo … so my husband knew that his chances were much worse. (R6)

Others said that they had experienced feelings of shock as, at diagnosis, they believed that they had been told the patient’s condition, although not curable, was treatable. This had led some relatives and patients to remain predominantly optimistic that a new treatment might stop their disease progressing:

[Haematologist] told us it wasn’t curable but it was treatable, it just always made us feel hopeful that they would treat it … [Haematologist] was so good at trying everything and anything. (R8)

In other cases, it was only after the patient’s (re)admission to hospital due to deterioration that discussions indicated treatment was failing and the patient was going to die imminently:

we got into the hospital, and then it was made clear … and we began to understand where we were going. (R3)

In such cases, relatives recalled being asked about preferred place of care and death, but described patients as being too ill to make considered decisions at this juncture. Moreover, options were limited if, for example, a hospice bed was required for terminal care, but not immediately available:

there wasn’t a hospice bed available. (R5)

One person had attended a multidisciplinary meeting to discuss her spouse’s preferences, but found the experience intimidating and overwhelming:

I found it incredibly daunting … you’re going as a relative stranger into a room full of people, who all know each other and … everybody is coming at it from all their different points of view … I just felt utterly bombarded and overwhelmed … it was horrible … I felt trapped … inside you’re screaming. (R5)

Another relative said that they would have appreciated earlier discussion concerning decreasing response to treatment and likelihood of death. Yet, despite attempts by haematologists, relatives portrayed most patients as reluctant to engage in end-of-life conversations, either with clinicians or with their family:

the earlier you discuss it, the better, frankly … when she firstly was told at the hospital of a 20% chance of survival, at that point, somebody ought to have come and had a discussion with her. (R9)

[Haematologist] was upfront … but he [patient] didn’t want to know. (R7)

He [patient] wouldn’t discuss it … he was frightened of dying … he was never good at dealing with anything emotive … he couldn’t come to terms with it. (R1)

Relatives described how late discussions resulted in patients returning home from hospital at short notice, with little time to organise support from relatives and/or community nursing services:

the consultant said … there was really no more [he/she] could do and [he/she] came into the [hospital] room and told him that … They did ask him where he wanted to go and he said, ‘I’d like to go home’ and there was a meeting between the GP surgery and various staff to try and have that happen … I was quite frightened at the thought of him coming home … because they [community nursing services] couldn’t promise any night care. (R10)

Doctors were said to be reluctant to predict prognosis after treatment cessation, and several relatives had been shocked and unprepared for how quickly death had ensued following hospital discharge:

they never said time is short … there was no weeks or months … they didn’t give us a time-span … and I wasn’t ready [for parent’s death]. (R2)

Relatives focussed on aspects of haematologists’ communication skills that had made end-of-life discussions easier or more difficult. An open, honest approach, with sufficient time for dialogue, was described positively, while an ‘abrupt’ attitude was perceived as disengaging:

we had every confidence in [haematologist, who] was excellent and explained everything beautifully. (R6)

it was abrupt … a bit full on for him [patient] … in your face … we’d only been in [the room] a minute or two and [the haematologist] just looked at him and said ‘Do you know where you want to die?’. (R1)

#### Family networks

Having relatives at home, or nearby, who could provide physical care and emotional support was considered essential for patients to remain at home. Some interviewees described themselves as too frail for this, while others were disinclined to carry out intimate tasks, seen as undignified for the patient, preferring instead to pay for assistance. Relatives experienced in delivering ‘hands-on’ nursing care said that they had felt confident in looking after the patient at home:

I’m not very big … I was just terrified that he would fall because I thought, if he falls, I’ll never get him up by myself. (R8)

having to change him, he was very embarrassed … you’ve got to think of the person’s dignity. (R10)

[paid carers] helped with bed bath and cleaned and tidied [patient]. (R3)

I was a carer … we did the course at college for palliative care … I’d been nursing for a long time … in the community. (R7)

The benefits of the wider family ‘pulling together’ to support the patient to remain at home were noted by many respondents: ‘family cohesion … the whole family worked at it’ (R3). Relatives who advocated for patients to remain at home at the end of their lives, if this was their choice, rather than being (re)admitted to hospital (see Theme 1), said that they did not feel their views were always given due consideration by the community health care team. Those with prior knowledge or experience of community nursing or hospice services remarked that this had helped them access services on behalf of patients. Others (e.g. R3 and daughter), who described themselves as ‘complete amateurs in a professional world’, relied on health care professionals to inform them about services:

I know how the system works … we got her fast tracked, equipment appeared … we got the Marie Curie nurse to stay on an evening and … I put a bit of emphasis on getting the Macmillan [nurse] on board … and she said, let’s take you to the day hospice, I’d like you to meet the consultant in palliative care … and everything was sorted out. (R2; community nurse)

he was not offered any chance to go into hospice … it would have been nice to have had the option … (R10)

Having prior contact with outpatient hospice services seemed influential in consideration of inpatient hospice care; but policy regarding the delivery of supportive care in this setting and the potential for this to vary between sites was said to restrict some patients accessing this resource:

we managed to get my mum to the day hospice, which she really enjoyed … and she agreed to admission to the hospice as well. (R2)

if his calcium had shot up and he needed more treatment, [hospice] wouldn’t have done it, they would have sent him back to hospital. (R6)

Several relatives mentioned feeling ‘let down’ by the lack of support for themselves as caregivers; only one person reported receiving written information to prepare them for what to expect at the time of their relative’s death:

some [nurses] are very good but some are not up to palliative care, ‘cos you’re not just there for the patient, you’re there for the relative as well. (R7)

I didn’t know what happens when people die … but the Marie Curie nurse left a little booklet … that was very, very helpful. (R6)

#### Resource availability

Adequate nursing support (e.g. district, Macmillan and Marie Curie nurses) was considered essential for relatives to provide care at home, and ‘gaps’, particularly overnight, were described as detrimental to relatives’ capacity to cope:

[home care] was never feasible unless we had much more support than we had, we’d have to have [had] almost full time support. (R4)

you’d have a job to get a nurse overnight. (R7)

GP involvement was also regarded as important. GPs who had been informed about the patient’s condition were said to have offered practical and emotional support to patients and relatives. However, the role of GPs could be constrained by poor or delayed communication with haematology staff:

[GP] came and said, ‘well, I think we’ve reached the end of the road now …’ I knew the hospital kept him informed while [patient] was seeing [haematologist] and [GP] said, ‘I can arrange for you to go into hospice, or if you’d rather stay at home, the choice is up to you’, and I said, ‘oh, he’ll stay at home please, if I can have some help to look after him’, and he said, ‘yes, I’ll arrange that for you’. (R8)

they don’t communicate with the GP surgeries, these specialist [haematology] centres. (R2)

Planning for, and timing of, hospital discharge was said to have a significant impact on resource availability and home death experiences. Advance planning, early involvement of hospital palliative care staff and timely liaison between service providers were linked with positive experiences:

the hospital got in touch with Macmillan … When [haematologist] stopped treatment [he/she] wrote to our GP and [GP] took charge … and told us what was available … We had a social worker come [who] got the care support forms going … carers coming in … we had Marie Curie come. (R3)

Contrastingly, relatives of patients discharged from hospital at short notice, when they were very close to death, reported insufficient time to organise support before the patient’s death; in some cases, patients did not survive long enough after treatment cessation to be discharged home. Access to hospice beds at short notice was also regarded as problematic:

they sent him home … and he died [next day] … [community support] should be sorted before [he] came home from the hospital. (R7)

there was a meeting between the GP surgery and various staff at the hospital to try and have that [hospital discharge] happen … but unfortunately [patient] didn’t reach that stage … (R10)

all these hospices are chock-a-block full, always. (R6)

### Theme 3: suggested changes to facilitate care and death in the preferred place

Participants proposed various changes to promote attainment of preferences. Early discussions of treatment cessation, prognosis and preferences were frequently mentioned:

it would have been a really good idea for someone to have [discussed prognosis] with her at a much earlier stage … it didn’t really happen at all. (R9)

Having adequate community support (nursing services, reliable round-the-clock help; GP involvement), for both patients and carers, was also considered essential if relatives were to cope satisfactorily at home and home death was to be achieved:

[the] Macmillan nurse was a gem … just telling us how to go about things … Marie Curie came to us about 4 times. (R3)

they could promise day care, but they couldn’t promise night care … (R10)

he collapsed in the bathroom and I just couldn’t lift him. (R1)

[the GP] got on to the district nurses … and he got a hospital bed … and hospice at home to help me. (R8)

the GP went out and did it [administered medication] at all hours … it was amazing … and just looked after the family. (R2)

Improved communication and co-ordination of services across secondary and primary care, as well as third sector organisations (e.g. hospice), was also considered important in ensuring that appropriate support was available:

if they want people to die at home … they need to get it together … I think that’s a big let-down, the communication between the hospital and the system outside. (R7)

no-one told me that he could have treatment at the hospice … I am sure we would have opted for hospice had we known that. (R10)

with the hospice, there needs to be a lot more interaction. (R1)

The final issue reported related to clinicians giving greater weight to relatives’ views on preventing hospital (re)admission of patients discharged home to die:

there was a real problem … with the out-of-hours service not understanding … and not being willing to listen … I accept that [the GP’s] first reaction might be yes, let’s get her into hospital, but [GP] should have stopped and listened … she died five days later at home. (R9)

## Discussion

### Main findings

Preferences were generally for death at home, but were not always achieved due to complex, interrelated factors, some amenable to change, others less so. Factors included the disease characteristics (e.g. the potential for sudden deterioration and death), occurrence and timing of discussions (e.g. treatment cessation, prognosis, place of care and death), family networks (e.g. the willingness/ability of relatives to provide care, their awareness of services and confidence to advocate on the patient’s behalf) and resource availability (e.g. clinical carers, hospice beds/policies). On reflection, some relatives considered hospital the ‘right’ place for the patient to have died. Others had shared strong preferences with patients for home death and acted to ensure this occurred. No patients died in a hospice, and a range of barriers were identified to this, notably lack of awareness about this facility, but also variation in service provision.

### Strengths and weaknesses

To our knowledge, this is the first study to examine preferred place of care and death in patients with blood cancers from the perspective of bereaved relatives. Qualitative methods suit the exploration of phenomena about which little is known^34^ and purposeful sampling facilitated the selection of ‘key informants’ who could provide rich description. Use of semi-structured interviews enabled relatives to focus on issues they considered significant. Our study sample, though comparatively small, is not atypical in qualitative research.^35^ Determining the ideal number of participants is dependent on various factors, including the quality of information obtained from interviewees.^27,36^ Participants in this study were the main care providers, so were inevitably well informed and able to relate rich, detailed data, which produced new and important insights. The nature of the subject, and the relatively short space of time between the death of their relative and the interview, meant recall was not an issue, although the possible influence of memory on the validity of findings cannot be completely dismissed. We recognise that the views of our study participants may not be reflective of the perspectives of the broader population of bereaved relatives;^37^ for example, the perceptions of relatives of younger patients, and those from minority ethnic groups, warrant further exploration.

### What this study adds?

Our study provides new insights into end-of-life care for people with haematological malignancies. While almost all relatives said that they had shared patient preferences for home care/death, some indicated that they had come to regard place of death as less important than achieving the best care for their relative. Participants in our study who had come to regard hospital as the ‘right’ place for the patient’s death said that this was due to the high-quality care provided by well-known staff, in familiar surroundings, and similar findings have been reported by others.^38^

Although a large body of research shows that home is generally the preferred place of death,^39,40^ many people do not die at home;^16^ for some, this may not be feasible or desirable.^41–43^ Interestingly, a recent population-based study of haematological cancer decedents found that more than 28% of those reporting a preference opted for hospital death.^44^ In such situations, hospital may represent a ‘safe haven’.^45^ Freedom from pain, having family present, not being a burden, having treatment choices followed and the opportunity to resolve conflicts have been ranked more highly by patients than actual place of death.^46^ Furthermore, review evidence indicates that what matters most to family members, regardless of the setting, is a good formal care team, who provide holistic ‘round-the-clock’ assistance, keep relatives informed and acknowledge the significance of the informal carer’s role.^47^

Regarding factors impacting on achievement of preferences, disease characteristics were important, with unpredictable pathways constraining or shaping choices, decisions and outcomes. Sudden, unanticipated deterioration was linked to increased likelihood of hospital death among patients having intensive hospital-based therapy, or receiving care at home. Relatives said that they would have welcomed guidance about the possibility of rapid change, as well as the signs of impending death. This issue has been highlighted by other authors,^48^ along with the difficulty of living in a state of permanent uncertainty about the way in which their relative’s disease might progress and the moment of death.^49^

Preoccupation with ‘doing the right thing’ when providing home care was widespread among our interviewees and was linked to feelings of stress and inadequacy. Other authors have described the ‘psychological complexity’^50^ relatives report as inherent in providing home care, due to high levels of responsibility, isolation and anxiety, as well as emotions such as anger, fear, frustration and sadness.^49^

Relatives in our study reported receiving varying levels of support from other family members and health professionals; those whose expectations for support had apparently not been met appeared to harbour enduring feelings of disappointment. Literature suggests that family home carers are sometimes viewed as a ‘co-workers’ by health care professionals and may not be identified as having their own needs; they may also be elderly, frail or ill themselves, requiring professionals to ‘think family’ when assessing their support needs.^47,51^

The occurrence and timing of end-of-life discussions was significant. A widespread preference was stated for such conversations to occur early and for them to be open, honest and frank. Accounts of undue optimism about treatment outcomes among patients, relatives and haematologists may be due to previous success with multiple lines of therapy and overly optimistic interpretation of communications with clinicians.^52^ Although maintaining some degree of hope, while remaining realistic, is a difficult balance,^53,54^ our findings underscore the need for clinicians to guard against raising false expectations.

There is growing consensus about the need for timely discussions^55^ and early shifts in treatment goals towards a palliative approach to care (including primary care involvement), as the likelihood of response or cure diminishes.^56,57^ Prognostication is notoriously difficult in haematology,^58^ however, which complicates clinician decisions regarding the ‘best’ time to initiate advance planning. Moreover, relatives in our study often highlighted patients’ reluctance to talk about death and dying, a finding that accords with studies showing that anxiety in such situations may trigger denial or avoidance behaviour.^59,60^ Reasons cited in the wider literature to explain haematologists’ delays in initiating conversations include the possibility of response to new treatments, time constraints, lack of confidence in communicating ‘bad news’ and disinclination to ‘abandon’ patients after prolonged treatments.^61,62^ Encouraging uptake of communication skills training among clinical staff could be one means of promoting supportive, honest conversations and increasing patient and clinician satisfaction.^63^

Relatives viewed the wider family network and dependable access to resources, including specialist palliative care and ‘round-the-clock’ community nursing services, as crucial to supporting patients to die at home. This matches findings from a recent systematic review^62^ and reportedly results in home death becoming a more realistic option.^64–66^ GP involvement was seen as facilitative of home death, due to the multifaceted role these clinicians exert within primary care. The need for more effective communication systems across the primary/secondary care interface was also identified, along with greater acknowledgement of caregivers’ feelings and preferences, which may also promote their recovery from grief.^67^

Hospice services were not accessed by the majority of our interviewees and factors said to influence this (unavailability of urgent or routine beds/ access, policies for administrating supportive treatments and prior family knowledge/contact) are reported elsewhere.^23,68^ A recent US study^69^ concludes that patients would benefit from closer collaboration between haematology and palliative care doctors and hospice access. Increased collaboration has been noted in the United Kingdom, alongside the need for clearer role definition, and consistent, flexible service provision.^24,61^

Issues requiring further research identified in our own and other studies include the following: the extent to which relatives participate in multidisciplinary meetings, and the support they receive/require for this;^70^ the interplay between relatives and patients whose preferences differ and the implications of this for hospital (re)admission;^71^ and GPs’ views of delivering end-of-life care to haematology patients and their educational requirements.^72^

## Conclusion

Our study highlights the factors that influence the attainment of preferred place of care and death in patients with haematological malignancy and their relatives. Although most people expressed a preference for home death, not all achieved this, due to interrelated factors, some of which were amenable to change, and others less so. Death in hospital may be preferred and appropriate for some, or considered the best option in hindsight.
